# Novel *FGFR1* and *KISS1R* Mutations in Chinese Kallmann Syndrome Males with Cleft Lip/Palate

**DOI:** 10.1155/2015/649698

**Published:** 2015-06-25

**Authors:** Hao Xu, Yonghua Niu, Tao Wang, Simin Liu, Hua Xu, Shaogang Wang, Jihong Liu, Zhangqun Ye

**Affiliations:** ^1^Department of Urology, Tongji Hospital, Tongji Medical College, Huazhong University of Science and Technology, Wuhan, Hubei 430030, China; ^2^Institute of Urology, Tongji Hospital, Tongji Medical College, Huazhong University of Science and Technology, Wuhan, Hubei 430030, China; ^3^Departments of Radiology, Tongji Hospital, Tongji Medical College, Huazhong University of Science and Technology, Wuhan, Hubei 430030, China

## Abstract

Kallmann syndrome (KS) is characterized by isolated hypogonadotropic hypogonadism (IHH) with anosmia and is sometimes associated with cleft lip/palate (CLP). In order to describe the clinical features, genetic etiology, and treatment outcome of KS males with CLP, we performed genetic screening for 15 known causal IHH genes (*KAL1*, *FGFR1*, *NELF*, *FGF8*, *CHD7*, *WDR11*, *SEMA3A*, *KISS1R*, *KISS1*, *PROKR2*, *PROK2*, *TAC3*, *TACR3*, *GNRH1*, and *GNRHR*) in four KS with CLP patients and six IHH patients without CLP. Two novel heterozygous missense mutations in *FGFR1*, (NM_001174066): c.776G>A (p.G259E) and (NM_001174066): c.358C>T (p.R120C), were identified in a 23-year-old KS male with cleft lip and an 18-year-old KS patient with cleft lip and palate, dental agenesis, and high arched palate, respectively. These two mutations were not presented in their healthy parents and 200 normal controls. One novel heterozygous missense mutation in *KISS1R*, (NM_032551): c.587C>A (p.P196H), was identified in an 18-year-old KS male with cleft lip and dental agenesis who developed sperm after being treated with gonadotropin. This mutation was also presented in his healthy father and grandfather. These results have implications for the diagnosis, genetic counseling, and treatment of KS and CLP males with mutations in *FGFR1* gene.

## 1. Introduction

Isolated hypogonadotropic hypogonadism (IHH) is a genetic disorder caused by a congenital defect in gonadotropin-releasing hormone (GnRH) secretion or action [1]. When IHH is combined with anosmia or hyposmia, it is considered Kallmann syndrome (KS), and it is considered normosmic IHH (nIHH) in the presence of a normal sense of smell [1]. A recent study in Finland showed that KS affects 1 : 30,000 in males and 1 : 125,000 in females [2]. Several genes have been implicated in IHH, including* KAL1*,* FGFR1*,* PROKR2*,* PROK2*,* NELF*,* FGF8*,* CHD7*,* WDR11*,* SEMA3A*,* KISS1R*,* KISS1*,* TAC3*,* TACR3*,* GNRH1*, and* GNRHR* [3]. Several nonreproductive phenotypes have also been described in KS patients, such as facial and oral developmental defects, synkinesia, hearing loss, and renal abnormalities [4].

Cleft lip/palate (CLP) occurs in 1 in 500–2,500 live births, and 30% of CLP cases are part of a syndrome with additional congenital anomalies. The remaining 70% of cases are isolated [5]. CLP and other facial and oral developmental defects, such as dental agenesis and high arched palate, have been described in KS patients. However, the genetic causes and treatment outcome of KS with CLP patients have not been well described.

This study aimed to perform clinical evaluation and observe the treatment outcome of four Chinese men with KS and CLP and explore their causative mutations.

## 2. Materials and Methods

### 2.1. Subjects

A total of 211 consenting male patients (age range, 18–36 year) at the andrologic clinic of Tongji Hospital, Tongji Medical College, Huazhong University of Science and Technology, were recruited into the IHH population. A total of 200 unrelated normal control males were randomly recruited from healthy individuals undergoing routine health examinations in the Tongji Hospital between June 2010 and September 2013. IHH was diagnosed based on the following criteria: at least 18 year old, incomplete or absent puberty, low serum testosterone levels, and low/normal gonadotropin levels, otherwise normal pituitary functions and normal hypothalamopituitary imaging findings. IHH patients exhibiting anosmia or hyposmia in the smell test were diagnosed with KS; otherwise, patients were diagnosed with nIHH.

Each patient's family history of pubertal development and associated nonreproductive phenotypes was obtained. Detailed physical examinations were performed, including testicular volume and penis length measurements using a Prader orchidometer and vernier caliper, respectively. Pubic hair and genitalia were evaluated according to Tanner pubertal stages. Olfaction was tested by the Alcohol Sniff Test [6]. In men able to ejaculate, a semen sample was analyzed using the World Health Organization (WHO) normal values based on the WHO 2010 reference limits [7]. The olfactory system was examined by brain magnetic resonance imaging (MRI). Renal structure and cryptorchidism were assessed by ultrasound. Patients were treated with human chorionic gonadotropin (hCG) and followed up for at least three years after their initial diagnosis.

The study protocol was approved by the ethics committee of Tongji Hospital, Tongji Medical College, Huazhong University of Science and Technology. Written informed consent was obtained from each participant after a full explanation of the purpose and nature of all study procedures.

### 2.2. DNA Extraction and Next-Generation Sequencing

Each participant's genomic DNA was extracted from whole blood using a DNeasy Blood and Tissue Kit (Qiagen, Hilden, Germany) according to the manufacturer's protocol. Fifteen known causal genes with the highest mutation frequency in IHH patients (*KAL1*,* FGFR1*,* PROKR2*,* PROK2*,* NELF*,* FGF8*,* CHD7*,* WDR11*,* SEMA3A*,* KISS1R*,* KISS1*,* TAC3*,* TACR3*,* GNRH1*,* GNRHR*) were selected, and a custom semiconductor targeted resequencing panel was designed as follows. Primers of 319 overlapping amplicons covering approximately 30 kb of coding sequence and flanking regulatory regions of each target gene were automatically designed with the Ion AmpliSeq Ready-to-Use custom designer platform (https://www.ampliseq.com/protected/dashboard.action). The primers (Life Technologies, Carlsbad, California, USA) which are able to perform ultrahigh-multiplex PCR reaction in one tube parallelly overlap 98.52% of the targeted region (30884 bp) by 319 amplicons. Ion Torrent adapter-ligated libraries were generated according to the Ion Ampliseq Library Kit 2.0 (Life Technologies, Carlsbad, CA, USA) protocol within about 5 hours. Briefly, 20 ng gDNA for every sample was quantitated by Qubit 2.0 fluorometer (Invitrogen, Carlsbad, CA, USA) for multiplex PCR amplification with the 2X primer pools. Then, the resulting amplicons were ligated to barcodes and Ion Torrent adapters (Life Technologies). Subsequently, we purified the libraries with AMPure XP beads (Beckman Coulter, Brea, CA, USA) and quantified the libraries by Qubit 2.0 fluorometer (Invitrogen, Carlsbad, CA, USA). Then, multiplexed barcoded libraries were enriched by clonal amplification using emulsion PCR on an Ion OneTouch 2 system (Life Technologies, CA, USA) according to the manufacturer's protocol. The emulsion PCR was performed as follows: the enzyme was activated at 99°C for 2 min. DNA was denatured at 99°C for 15 seconds, and annealing and extension were performed at 60°C for 4 min for 17 cycles. Massively parallel semiconductor sequencing was performed on Ion 318 Chips using the Ion PGM Sequencing 200 v2 Kit (Life Technologies, CA, USA) and a Personal Genome Machine Sequencer (Ion Torrent) according to the manufacturer's instructions.

### 2.3. Bioinformatic Analysis

Data outputs obtained by semiconductor sequencing were processed using Torrent Suite version 3.6.2 (Life Technologies, Carlsbad, CA, USA) to generate sequence reads, trim adapter sequences, remove poor signal-profile reads, align to the hg19 human reference genome, analyze coverage, and call variants. Detected variants were then annotated with Ion Reporter (https://ionreporter.lifetechnologies.com/ir) and filtered using dbSNP137, Hapmap, and 1000-Genome Project. Functional prediction and conservation analysis were performed using ANNOVAR.

### 2.4. Sanger Sequencing Validation

Only the novel variants that had not been previously described in dbSNP137, 1000-Genome Project, and were predicted to cause nonsynonymous coding changes were sequenced by Sanger sequencing to validate whether these mutations detected by PGM are true mutations. Briefly, PCR amplification of the DNA region across the variants in the patients who carried the variants was performed using Taq Hot Start Version Kit (Takara Bio, Otsu, Shiga, Japan) and specific primer (see Supplementary Table 1 in Supplementary Material available online at http://dx.doi.org/10.1155/2015/649698) according to the manufacturer's protocol. The Big Dye v.1.1 Terminator cycle sequencing kit and Applied Biosystems 3500xl capillary sequencer (Applied Biosystems, Foster, CA, USA) were used with individual forward and reverse primers (Supplementary Table 1) to detect any possible false positive errors. In order to determine whether the true mutations were presented in the 200 normal controls, we performed PCR amplification of the DNA region across the true mutations using Taq Hot Start Version Kit (Takara Bio, Otsu, Shiga, Japan) and specific primer (Supplementary Table 1) in 200 normal controls samples. Then, Sanger sequencing was performed in the PCR products with the specified primers. Similarly, we sequenced the DNA regions across the true mutations by Sanger sequencing in the family members of each patient who harbored true mutation to find weather these mutations were presented in the other family members. The technically uncovered 456 bp regions of each of the nine targeted genes and amplicons with less than 50X coverage were also directly sequenced by Sanger sequencing. The oligonucleotides used for amplification are shown in Supplementary Table 2.

## 3. Results

### 3.1. Clinical Characteristics

A total of 4 KS patients with CLP were identified in a cohort of 211 Chinese IHH patients including 99 KS patients and 112 nIHH patients. The clinical features and follow-up results for each of the 4 KS males with CLP are summarized in Table 1.

### 3.2. Next-Generation Sequencing

**Figure 1:**
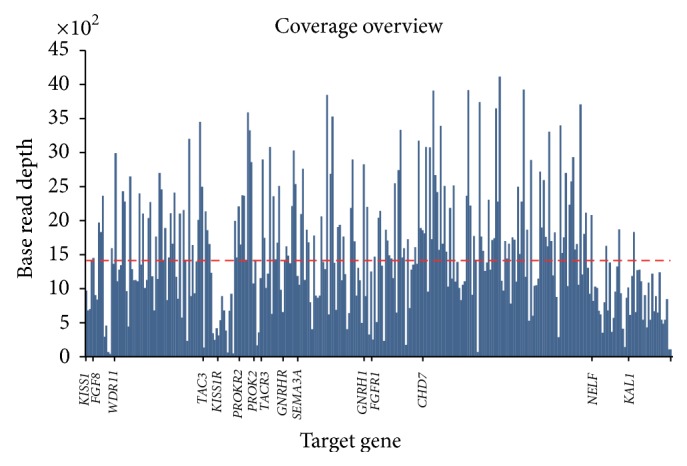
The sequencing coverage of the 15 genes from the ten samples. Blue graphs represent the distribution of coverage of 15 IHH genes from 10 samples. The dashed line is the mean coverage (1402X) of the 15 IHH genes.

Using a custom panel, we performed semiconductor sequencing in 4 KS males with CLP and 6 IHH/KS males without CLP (including five KS patients and one nIHH patient). The average output was 504007 mapped reads per sample, and 99.4% of the sequence reads were mapped to targeted gene regions. The distribution of reads across the 319 amplicons was consistent across samples, with an average uniformity of amplicon coverage of 93.1%. The 1X, 10X, and 100X base coverage were 99.9%, 99.6%, and 97.3%, respectively. The mean uniformity of base coverage was 92.8%. The average base coverage depth in the 30 kb target region was 1402X across all samples (Figure 1). We identified 327 total variants with an average of 33 variants per patient (range 17~43 variants) (Supplementary Table 3). We excluded variants presented in dbSNP137 or the general population with a minor allele frequency greater than 5% according to the 1000-Genome Project, intronic variants, and synonymous exonic variants, leaving 21 remaining variants (Supplementary Table 4). These mutations were predicted to cause nonsynonymous coding changes in seven genes, with an average of three variants per patient (range 0–5 variants).

### 3.3. Sanger Sequencing Validation

**Figure 2:**
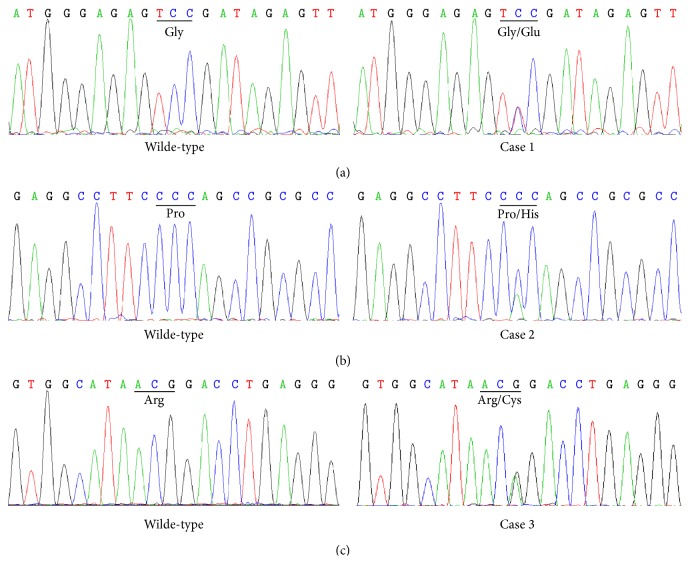
Mutations in three Kallmann syndrome (KS) males with cleft lip/palate. Heterozygous missense mutations in (a)* FGFR1*, (NM_001174066): c.776G>A (p.G259E) in case 1, (b)* KISSR1*, (NM_032551): c.587C>A (p.P196H) in case 2, and (c) FGFR1, NM_001174066: c.358C>T (p.R120C) in case 3. For comparison, normal sequences of the corresponding regions are indicated.

We performed Sanger sequencing to rule out false positives and confirmed that 3 of the 21 variants (Table 2) were true mutations. There were no additional mutations in the 456 bp regions of each target gene or in amplicons with less than 50X coverage. In summary, two novel heterozygous missense mutations in* FGFR1*, (NM_001174066): c.776G>A (p.G259E) and (NM_001174066): c.358C>T (p.R120C) (Figure 2), were identified in a 23-year-old KS male with cleft lip and an 18-year-old KS patient with cleft lip and palate, dental agenesis, and high arched palate, respectively. These two mutations were not presented in their parents and other heathy family members. One heterozygous missense mutation in* KISS1R *which was reported previously, (NM_032551): c.587C>A (p.P196H) (Figure 2), was identified in an 18-year-old KS male with cleft lip and dental agenesis. However, it was presented in the healthy father and grandfather of this patient. We did not identify these three mutations in any of the 200 control subjects. Furthermore, we did not identify any mutations in the six IHH/KS patients without CLP.

## 4. Discussion

Because the clinical features and genetic etiology of KS patients with CLP have not been well described, we performed clinical and genetic analysis in Chinese KS males with CLP. In order to do the genetic analysis in a fast and cost-effective way, we used a next-generation sequencing strategy by the PGM sequencer. Using this strategy, we screened 15 selected IHH/KS casual genes (*KAL1*,* FGFR1*,* PROKR2*,* PROK2*,* NELF*,* FGF8*,* CHD7*,* WDR11*,* SEMA3A*,* KISS1R*,* KISS1*,* TAC3*,* TACR3*,* GNRH1*,* GNRHR*). Each of the 15 selected causal genes accounts for a certain percentage (*KAL1*: 5%–10% [8];* FGFR1*: 10% [8];* PROKR2*: 5% [9];* PROK2*: 1% [9];* NELF*: 1.8% [10];* FGF8*: 1% [9];* CHD7*: 6% [8];* WDR11*: Unknown;* SEMA3A*: 6% [11],* KISS1R*: <5% [12];* KISS1*: <2% [12];* TAC3*: <1%,* TACR3*: 5-6% [8];* GNRH1*: 1% [9];* GNRHR*: 4% [8]) of the IHH patients. A recent study showed that when 13 most common IHH/KS genes (*KAL1*,* GNRHR*,* FGFR1*,* KISS1R*,* TAC3*,* TACR3*,* FGF8*,* PROKR2*,* PROK2*,* CHD7*,* NELF*,* GNRH1*, and* WDR11*) are studied, 54.2% the IHH patients had a mutation in at least one gene [13]. In our study, we identified 3 mutations in three of the 10 patients, respectively, after being filtered agianst the public database and validated by Sanger sequncing. One heterozygous missense mutation in* KISS1R*, NM_032551: c.587C>A (p.P196H), which was reported previously, and two heterozygous novel missense mutations in* FGFR1*, NM_001174066: c.776G>A (p.G259E) and NM_001174066: c.358C>T (p.R120C), were identified. These three missense mutations were predicted to be damaging in functional prediction by SIFT and PolyPhen2. Conservation analysis using PhyloP and GERP++ shows that all of them are highly conserved (Table 2).

**Figure 3:**
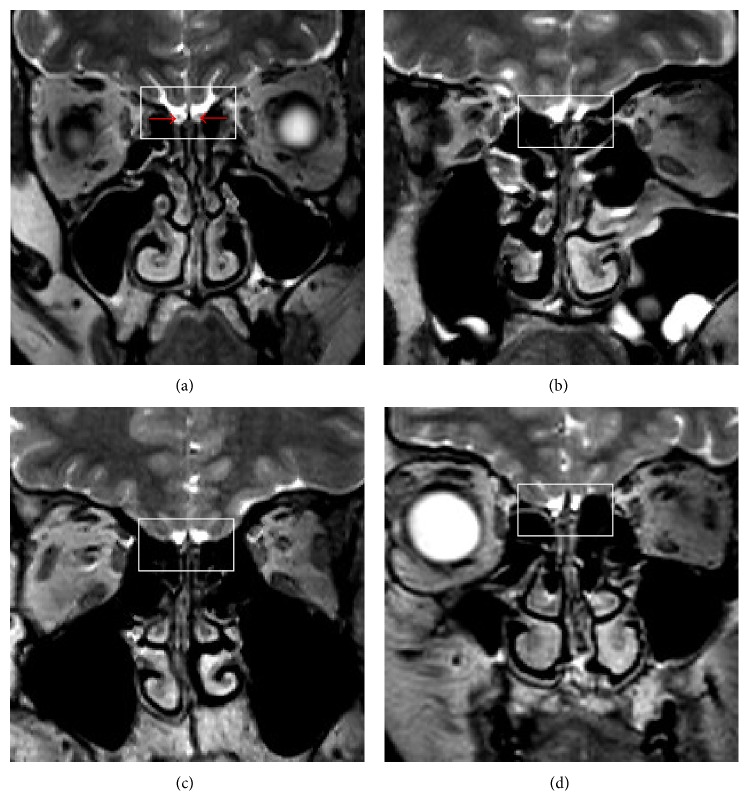
Magnetic resonance imaging (MRI) results of the three Kallmann syndrome males with cleft lip/palate. (a) T2-W MR images show normal structures in a normal control male. The red arrows indicate olfactory bulbs. ((b), (c), (d)) The MRI showed the lack of the bilateral olfactory bulb, the olfactory tract, and sulcus (squares) in cases 1, 2, and 3, respectively.

Fibroblast growth factor receptor 1 (*FGFR1*) encodes one of four FGFRs, which are tyrosine kinase family cell surface receptors. They function in the olfactory system and GnRH ontogeny was consistent with a role in the KS patient phenotype [14].* FGFR1* mutated in as many as 10% of all KS cases and patients harboring* FGFR1* mutations exhibited a broad spectrum of associated nonreproductive phenotypes, including CLP and dental agenesis [14]. It has been reported that KS linked to* FGFR1 *with or without CLP is transmitted as an autosomal dominant disease [12, 14]. We identified two* FGFR1* mutations in two of the four KS patients with CLP. The parents of the two patients had a normal phenotype and no other family members were affected by this disease (Supplementary Figure 1). Both patients exhibited anosmia and bilateral absence of the olfactory bulbs and tracts by MRI (Figure 3), consistent with complete KS. One patient also had dental agenesis and a high arched palate. This phenotype heterogeneity of KS caused by* FGFR1* mutations in our patients is consistent with previous report [4]. To our knowledge, this is the first report of an* FGFR1* mutation in KS male with a high arched palate. Besides, the c.776G>A mutation was not presented in the normal parents of case 1 and the c.358C>T mutation was not presented in the normal parents and old sister of patient 3 (data not shown), suggesting that these mutations are novel* de novo* mutations. Our data supports that KS with CLP linked to* FGFR1* is transmitted as an autosomal dominant disorder which has been reported previously [14].

The* KISS1R* gene encodes the G protein-coupled receptor GPR54, which regulates GnRH secretion. Studies have shown that* KISS1R* loss-of-function mutations contribute to nIHH by affecting GnRH secretion, but not olfactory bulb development or GnRH neuronal migration [15, 16]. Relatively few* KISS1R* mutations have been reported in IHH patients to date, and all reported mutations are compound heterozygous or homozygous mutations [17–22]. Here, we identified one heterozygous* KISS1R* mutation in a KS patient with IHH, anosmia, lack of bilateral olfactory bulbs (Figure 3), cleft lip, and dental agenesis. This mutation was also presented in the normal father and grandfather of this patient (Supplementary Figure 2). Interestingly, this mutation has been previously reported in a girl with central precocious puberty [23]. This evidence suggested that this mutation may not be a pathogenic mutation in KS patient though functional prediction indicates the c.587C>A mutation in* KISS1R* causing damage to the KISS1R. However, in vitro functional studies are needed to confirm the consequence of this mutation. Besides, we did not detect any mutations in the other known causal KS genes, including* KAL1*,* FGFR1*,* NELF*,* FGF8*,* CHD7*,* WDR11*,* SEMA3A*,* PROKR2,* and* PROK2*.

In the present study, we observed that the testicular size and length of penis did increase in the cases 1, 2, and 3. These can be explained as results of the normalized serum testosterone levels in these three patients after being treated with hCG. The serum testosterone levels of case 4 remained in low level, so no obvious increase of the testicular size and phallus length was observed. Furthermore, the hCG treatment succeeded in inducing spermatogenesis in the* KISS1R* mutation patient. However, it failed to result in sperm in the ejaculation of the two* FGFR1* mutation patients (Table 3). Our data showed that the heterogeneity response of testis to the hCG treatment existed. Recent studies may cast new light on the mechanism of this heterogeneity. It has reported that* KAL1* gene, which plays a key role in the GnRH neuron migration by regulating FGFR1 signaling activation, is expressed in the marsupial gonad and participates in gametogenesis directly within the gonads themselves [24]. More recently, some authors reported that Fgfr1 was expressed in the entire population of undifferentiated spermatogonia in mammalian testes and FGF8 signal positively regulated the numbers of undifferentiated spermatogonia through* FGFR1* [25]. Based on these data we predict that mutations in the* FGFR1* may affect the spermatogenesis outcome in the testes of the KS patients after being treated with gonadotropin. However, more studies should be done in the future to prove the association with this phenotype.

## 5. Conclusions

We identified two novel* FGFR1* mutations in two of the four KS patients with CLP in this study. The results have implications for the diagnosis, genetic counseling, and treatment of KS and CLP males with mutations in* FGFR1* gene.

## Supplementary Material

The short description (in paragraph style) of the Supplementary Material is:
Supplementary table 1: The primers used for amplification of genomic region across the novel missense variants detected by next generation sequencing are listed in this table.
Supplementary table 2: The primers used for amplification of the technically uncovered 456 bp regions of each of the nine targeted genes and amplicons with less than 50X coverage in the next generation sequencing are listed in this table.
Supplementary table 3: Summary of the sequencing results of the ten samples after sequenced by next generation sequencing are listed in this table.
Supplementary table 4: The 21 remaining variants after filtered Sanger sequencing validation are listed in this table. Note that only three mutations were confirmed to be true mutations by Sanger sequencing.
Supplementary figure 1: The Pedigree of the four KS and CLP patients are presented in this figure. Note that the father of case 4 also had CLP but normal puberty.
Supplementary figure 2: The mutation in KISS1R, (NM 032551): c.587C>A (p.P196H) was presented in the healthy father and grandfather of case 2.

## Figures and Tables

**Table 1:** Summary of clinical assessment of the four Kallmann syndrome males with cleft lip/palate.

Case no.	Age at diagnosis (yr)	Clinical diagnosis	Inheritance	Olfactory	Olfactory bulbs on MRI	Renal findings on ultrasounds	Other features
1	23	KS	Sporadic	Anosmia	Absence (B)	Normal	Cleft lip, gynecomastia
2	18	KS	Sporadic	Anosmia	Absence (B)	Normal	Cleft lip, dental agenesis
3	18	KS	Sporadic	Anosmia	Absence (B)	Normal	Cleft lip and palate, dental agenesis, high arched palate
4	18	KS	Familial	Anosmia	Absence (B)	Normal	Cleft lip

B, bilateral; CLP, cleft lip/palate.

**Table 2:** Novel mutations detected by semiconductor sequencing and confirmed by Sanger sequencing.

Case no.	Position	Gene	Type	Zygosity	Nucleotide substitution	Consequence	Novelty	Sanger sequencing validation	SIFT score	PolyPhen result	PhyloP score	GERP++ score
1	38279353	*FGFR1 *	SNV	Het	C/T	Nonsynonymous	Novel	TURE	0	D	2.542	5.4
2	919955	*KISS1R *	SNV	Het	C/A	Nonsynonymous	Previously reported	TURE	0	D	2.099	4.6
3	38283760	*FGFR1 *	SNV	Het	G/A	Nonsynonymous	Novel	TURE	0	D	2.737	5.78

**Table 3:** Biochemical and clinical characteristics of the four Kallmann Syndrome men with cleft lip/palate at baseline and followup.

Case no.	Heigt (cm)		LH (mIU/mL)		FSH (mIU/mL)		Testosterone (ng/mL)		Mean Testicular Volume (mL)		Penis length (cm)		Tanner stage of Pubic hair		Sperm Count (million/mL)		Treatment	Duration of treatment (months)
	B	F	B	F	B	F	B	F	B	F	B	F	B	F	B	F		
1	182	185	0.97	0.48	0.92	0.30	0.15	2.03	2.0	10.0	2.5	6.0	1	5	ND	0	hCG	42
2	172	175	1.18	0.03	0.62	0.20	0.23	5.65	4.0	20.0	3.0	6.5	1	5	ND	11.92	hCG	50
3	171	176	<0.01	0.38	0.82	0.24	0.29	2.45	2.5	12.0	2.5	7.0	1	5	ND	ND	hCG	36
4	172	175	0.2	0.23	0.5	0.71	0.16	0.18	2.5	4	5	5	3	3	ND	0	hCG	38

B, baseline; F, followup; ND, not done.
