# Supplementary figures and images for: Novel FGFR1 and KISS1R Mutations in Chinese Kallmann Syndrome Males with Cleft Lip/Palate
Source: Biomed Res Int. 2015 Jun 25;2015:649698. doi: 10.1155/2015/649698 (PMC4496468; doi:10.1155/2015/649698)

## Slide 1
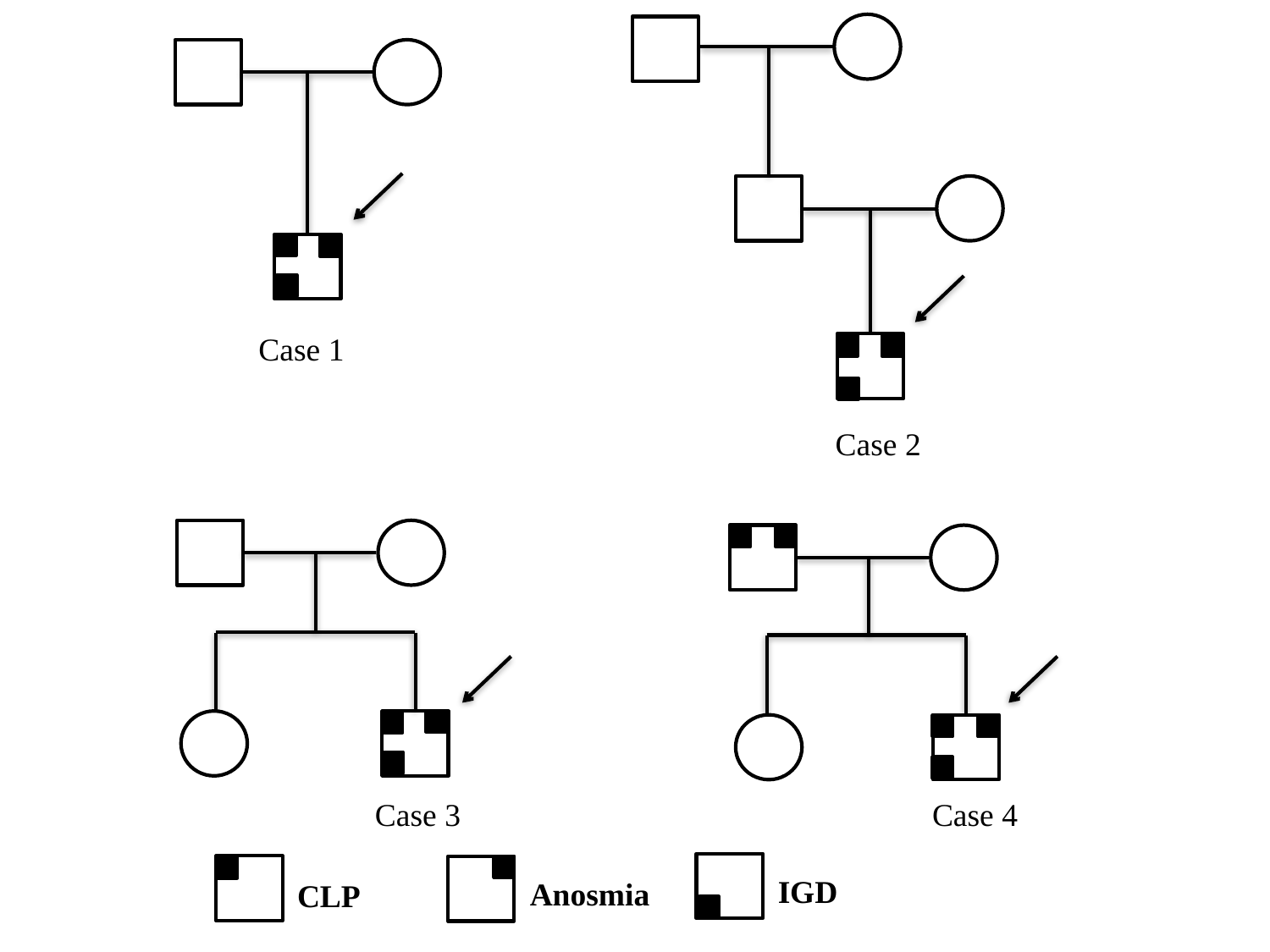

Case 1
Case 2
Case 3
Case 4
IGD
Anosmia
CLP

Supplement: Supplementary file 1 — The short description (in paragraph style) of the Supplementary Material is: Supplementary table 1: The primers used for amplification of genomic region across the novel missense variants detected by next generation sequencing are listed in this table. Supplementary table 2: The primers used for amplification of the technically uncovered 456 bp regions of each of the nine targeted genes and amplicons with less than 50X coverage in the next generation sequencing are listed in this table. Supplementary table 3: Summary of the sequencing results of the ten samples after sequenced by next generation sequencing are listed in this table. Supplementary table 4: The 21 remaining variants after filtered Sanger sequencing validation are listed in this table. Note that only three mutations were confirmed to be true mutations by Sanger sequencing. Supplementary figure 1: The Pedigree of the four KS and CLP patients are presented in this figure. Note that the father of case 4 also had CLP but normal puberty. Supplementary figure 2: The mutation in KISS1R, (NM 032551): c.587C>A (p.P196H) was presented in the healthy father and grandfather of case 2. [file 649698.f1.zip › Supplementary_figure_1_2738_1383944.pptx]
